# Blood Loss Estimation in Small Animals and Assessment of a Pictorial Tool to Improve Accuracy in a Global Population of Veterinary Anesthesia Staff

**DOI:** 10.3389/fvets.2020.00212

**Published:** 2020-05-07

**Authors:** Scott H. Cumming, Fernando Martinez-Taboada

**Affiliations:** Anaesthesia Department, The Veterinary Teaching Hospital Sydney, The University of Sydney, Camperdown, NSW, Australia

**Keywords:** blood loss, blood loss estimation, hemorrhage, pictorial guide, anesthesia

## Abstract

Visual estimation of blood loss is the most common form of evaluating intraoperative hemorrhage, and is also the most inaccurate. This study investigated the visual estimation accuracy of a global population of anesthesia staff and students as an initial estimation and also with the assistance of a pictorial guide. A voluntary, two-part, online, anonymous survey was distributed to members of two email databases with an interest in anesthesia, including students, nurses, interns, residents, general practitioners, and specialists. The survey consisted of visual and brief descriptive depictions of blood loss scenarios involving small animals, principally including images of common surgical items and receptacles containing a blood-like substance. Each participant estimated the blood volume (in mL) for each scenario twice, initially (Pre-Guide [PGD]) and then with the aid of a pictorial guide (With-Guide [WGD]). The pictorial guide used similar images labeled with corresponding volumes. Data was analyzed for normality with the Shapiro-Wilks test, corrected to absolute error and compared for statistical significance using the Wilcoxon signed-ranks test or the Kruskal-Wallis test as appropriate. The overall raw PGD phase median estimation error was−27 mL (range −99 to 248 mL). The PGD raw median error increased with scenario complexity. There were no differences between role, gender, experience, or country of origin. The overall median raw estimation error for the WGD phase was 13 mL (range −80 ml to 143 mL) (*p* = 0.0128). Visual blood loss estimation is inaccurate amongst veterinary anesthetists and associated staff, showing decreasing accuracy with increasing complexity. A pictorial guide improves the accuracy generally, and specifically for more complex scenarios which are likely to reflect the clinical situation.

## Introduction

Acute hemorrhage impairs oxygen delivery and rapid management improves outcomes (1, 2). In small animals, with low total blood volumes, small amounts of blood loss can logically have significant deleterious effects. Traditional systemic parameters alone, including mean arterial pressure (MAP) and heart rate (HR), have been shown to have poor correlation with accurate hemorrhage assessment (3). Accurate estimation of intraoperative blood loss is an important component of circulatory competency assessment and management (4, 5). Estimating blood loss volume, together with systemic indicators such as digital pulse pressure (dPP) and MAP, provides important information for making appropriate fluid therapy decisions including whole blood transfusion (6, 7).

In the medical literature, research has been particularly focused around obstetrics, where hemorrhage is common. Visual estimation has been found to be both the most common method of estimation and inaccurate, with both over- and underestimation and with wide ranges (5, 8). Recent medical studies have focused on development of pictorial guides to aid in the estimation of blood loss, generally with findings of improved accuracy (9–11). In the veterinary literature there has been little research into estimating blood loss. Colorimetric methods, where spectrophotometrically-measured hemoglobin concentration is used to estimate blood volume, have been positively correlated with direct-measurement in two studies (12, 13). However, these methods are both time consuming and technically challenging. Our previous study investigated the accuracy of blood loss estimation and the utility of a pictorial guide in a university setting, finding that the pictorial guide improved accuracy (14).

Our objective in this study was to investigate the visual estimation accuracy of a global population of veterinarians, associated staff and veterinary students with an interest in anesthesia, and evaluate the utility of a pictorial guide for the improvement of visual estimation. Our primary hypothesis was that visual estimation would be poor and a pictorial guide would improve visual estimation of blood loss in small animals. We also hypothesized that more complex estimation requirements would result in more inaccuracy.

## Materials and Methods

This project was approved by the University of Sydney Human Research Ethics Committee (number 2018/633).

Images depicting blood collected in various receptacles were developed using artificial blood according to a process reported in detail previously (14). Briefly, these images were used to represent scenarios of blood loss in surgical situations involving small animals. These scenarios also included background information which matched the volumes of blood represented. The scenarios were designed so that they successively increased in complexity, whereby each scenario comprised of more images, or images that were more challenging to estimate, than the preceding scenario. The scenarios consisted of: (1) a suction pot containing 66 mL of artificial blood; (2) a kidney dish containing 105 mL of artificial blood; (3) a puddle of 50 mL and swab with 7 mL of artificial blood; (4) a puddle of 17 mL and laparotomy sponge with 40 mL of artificial blood; and (5) a laparotomy sponge with 100 mL, a swab with 6 mL, and a swab with 3 mL of artificial blood. Similar images created in the same manner were also used to create a pictorial guide (the “Guide”) with labeled volumes and was hosted on the online platform Wix (wix.com, Tel Aviv, Israel). Both the scenarios and the Guide are displayed in Appendices A, B respectively.

A survey was created and hosted online using Survey Monkey (surveymonkey.com, San Mateo, CA, USA). The survey, written in English, consisted of five sections and complied with the guidelines detailed in the “Checklist for Reporting Results of Internet E-Surveys (CHERRIES)” (15). The first section provided a summary of the Participant Information Statement as well as information on storage of the data and an “opt in” selection was required before accessing the actual survey. The second section gathered demographic and professional information including role and years of experience. The third section consisted of the five “scenarios,” displaying an image or images and the background information, giving information as to the size of the item or receptacle, but without the volume of blood, and included a text input box. The fourth section provided a link to the online Guide on a separate browser tab. The final section repeated the scenarios from section three in the same order with a similar text input box. All scenarios appeared in the same order for both phases for all respondents. An informal trial of the survey was conducted to confirm functionality prior to distribution.

An email was sent to the American College of Veterinary Anesthesia and Analgesia email database list (ACVAA, 1,472 members) and the Sociedad Espanola de Anestesia y Analgesia Veterinarias (SEAAV, 310 members) with a link to the online survey, inviting participants to complete the survey. The survey was “open” to all recipients of the email with the link (15). Potential participants were informed in the introductory email of the purpose of the research, the expected length of the survey and provided with a link to the Participant Information Statement. No incentives were offered for participation. In accordance with CHERRIE Guidelines, all information was anonymous and no identifying data was collected (15). The survey was open for ~12 weeks. At the conclusion of the collection period, all of the data was downloaded from the Survey Monkey site and reviewed for obvious duplication and data integrity.

Respondents progressed through the survey by assessing each scenario and inputting an estimated volume of blood as a numerical value (in mL). Respondents initially assessed each scenario with only the information presented in the survey, and then assessed each scenario again with the aid of the Guide. In this way, each respondent provided one set of responses prior to the Guide [Pre-Guide (PGD)] and one set of responses using the Guide [With-Guide (WGD)]. All respondents completed the survey in a single session. Review and alteration of responses was possible within the sections (PGD and WGD), but not between the sections once the participant had progressed to the next section.

### Statistical Methods

A sample size calculation using 90% power and 5% significance level for a 10 mL difference between responses resulted in a minimum size of 36.

The given responses were converted into a “raw” error value by subtracting the actual scenario volume from each estimated value, resulting in comparability between scenarios and a measure of over- or under-estimation. The “raw” error values were also converted into “absolute” values by removing the negative sign to remove the confounding effects on the median of negative values, in order to better evaluate the magnitude of the deviation of the estimate from the actual volume.

Descriptive statistics were used to assess the data initially and normality tested using the Shapiro-Wilk test. The statistical difference between paired groups of data was tested with the Wilcoxon signed ranks test. Comparison between multiple groups, including role groups and scenarios, was made using the Kruskal-Wallis test and Dunn's test with the Bonferroni correction. Both “raw” and “absolute” values were analyzed. Comparison between the PGD and WGD phases for both Groups and Scenarios was conducted only for complete data sets containing both PGD and WGD values.

Significance levels were set at *p* < 0.05. Values are reported as median (range), negative raw error values indicate underestimation, while all other values are considered overestimation. All analyses and calculations were undertaken in RStudio Version 1.1.463 for Mac OS 10.14.4 (The R Foundation for Statistical Computing, Austria).

## Results

### Population Data

Of the 1,782 recipients of the email inviting participation, 215 respondents viewed the first page giving a view rate of 12 %. Of the 215, 178 went on to participate, giving a participation rate of 82.7%. One hundred and thirty four respondents completed the entire survey for a completion rate of 75%.

Data was excluded if the integrity could not be guaranteed. For example, where the inputted value was improbably small with respect to the given scenario and likely indicated an input error, the entire set of responses was excluded. Of the 178 respondents, 21 data sets were excluded for this reason, resulting in 157 data sets, both complete and incomplete. On two occasions it was clear that a typographical error had been made in the input of a response wherein the value ended in two consecutive “0's” and was improbably large (i.e., larger than the entire blood volume of the species in the scenario). On these occasions only, the value was corrected by removing the second “0” and retained. After exclusions, the data retained for analysis included a total of 157 PGD and 126 WGD respondents, resulting in 730 and 685 observations respectively. Respondents who completed both phases reported 15 different countries of origin. Years of experience ranged from “student” to “>15” years (median “10 to 15” years).

The General Practitioner and Advanced General Practitioner categories were combined into a single group, and the Student and Intern categories were combined into a single group due to low respondent numbers.

### Pre-guide Phase

#### Overall

The overall raw PGD phase median estimation error was 27 mL less than the actual volume, with a range of 99 mL less-than the actual volume to 248 mL more-than the actual volume.

The overall absolute PGD phase estimation error was 34 mL (range 0 to 243 mL).

There were no differences between the estimation errors for respondents when grouped according to country of origin, gender, or years of experience.

#### Role

All role groups underestimated the volume in the PGD phase. The Specialist group had a smaller raw estimation error than the General Practitioner group (*p* = 0.051). There was no difference between all other role groups for raw PGD estimation error, and no differences between any role group for absolute estimation error. All results are reported in Table 1.

**Table 1:** Group results displayed in mL as median and range for pre-guide (PGD).

	**PGD 157 data sets**		
**Group**	***n***	**Obs**.	**PGD (raw)**
			**PGD (absolute)**
All	157	785	−27 (−99 to 243)
			34 (0 to 243)
General Practitioner	28	140	−31 (−97 to 141)^*^
			36 (1 to 141)
Student/	26	130	−27 (−89 to 191)
Intern			34 (1 to 191)
Nurse/	16	80	−27 (−89 to 99)
Technician			32.5 (1 to 99)
Resident	23	115	– 27 (−99 to 243)
			37 (1 to 243)
Specialist	64	320	−17 (−95 to 241)^*^
			34 (o to 241)
*p*			^*^0.051

* *Denotes significance between groups*.

#### Scenarios

There were differences between the raw estimation errors for several scenarios. Scenario 3 had a greater raw estimation error than both Scenario 1 (*p* = 0.0008), Scenario 2 (*p* < 0.0001), and Scenario 4 (*p* = 0.0006). Scenario 5 had a greater raw estimation error than Scenario 1 (*p* = 0.0002), Scenario 2 (*p* < 0.0001), and Scenario 4 (*p* = 0.0001).

Scenario 5 had a greater absolute estimation error than Scenario 1, Scenario 2, Scenario 3, and Scenario 4 (*p* < 0.0001).

### With-Guide Phase

#### Overall

The overall median raw estimation error for the WGD phase was 13 mL above the actual volume with a range of −80 ml to 143 mL. The median absolute estimation error for the WGD phase was 20 ml (range 0 to 143 mL).

There were no differences between the respondents when grouped according to gender or years of experience or nominated country.

#### Role

All roles groups overestimated the volume in the WGD phase. The General Practitioner group had a smaller estimation error than the Student/Intern group (*p* = 0.0128). All other groups were not different (*p* > 0.05). There were no differences between the role groups for the absolute error in the WGD phase.

#### Scenarios

Both scenarios 1 and 2 had a greater raw median WGD error than Scenario 3 (*p* < 0.0001), Scenario 4 (*p* = 0.0001), and Scenario 5 (*p* < 0.0001). Scenario 1 and Scenario 2 were not different to each other.

Scenarios 1 and 2 had a greater absolute median WGD error than Scenarios 3 (*p* < 0.0001), 4 (*p* < 0.0001), and 5 (*p* < 0.0001).

Scenarios 3, 4, and 5 were not different to each other for raw or absolute error values.

### Comparison Pre-guide Phase to With-Guide Phase

#### Overall

**Figure 1:**
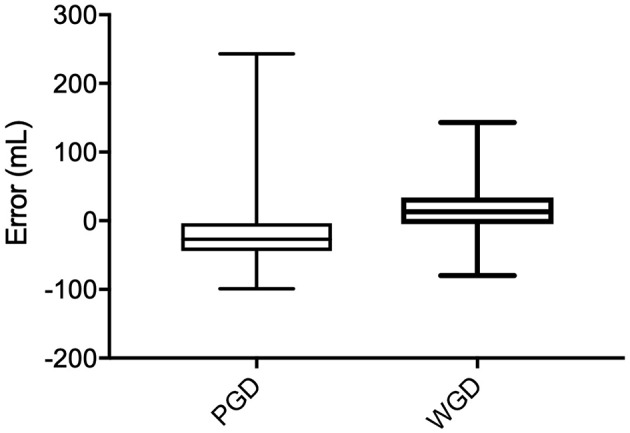
Raw error (mL) comparison between Pre-Guide (PGD) and With-Guide (WGD) for each role group.

Only complete data sets were compared between PGD and WGD phases. There was a difference between both the overall raw and overall absolute estimation error between the PDG phase and the WGD phase (*p* < 0.0001) (Figure 1).

#### Role

All of the role groups showed a difference between the PGD and WGD phases and all had a reduced median error in the WGD phase compared with the PGD phase for both raw values (*p* < 0.0001), and the absolute values (*p* < 0.05). See Table 2.

**Table 2:** Group results displayed in mL as median and range for 126 data sets Pre-Guide (PGD) and With-Guide (WGD) as raw and absolute values.

			**PGD (126)**	**WGD**	
**Group**	***n***	**Obs**.	**PGD (raw)**	**WGD (raw)**	***p***
			**PGD (absolute)**	**WGD (absolute)**	
All	126	630	−27 (−99 to 243)	13 (−80 to 143)	<0.0001
			34 (0 to 243)	20 (0 to 143)	<0.0001
General Practitioner	16	80	−36 (−94 to 95)^*^	8 (−80 to 95)^*^	<0.0001
			37 (3 to 95)	21 (0 to 95)	<0.0001
Student/	24	120	−27 (−83 to 191)	18 (−37 to 141)^*^	<0.0001
Intern			33 (1 to 191)	21.5 (1 to 141)	0.0004
Nurse/	10	50	−27 (−70 to 91)	16.5 (−27 to 54)	<0.0001
Technician			31.5 (2 to 91)	19 (0 to 54)	0.0077
Resident	21	105	– 27 (−99 to 243)	8 (−79 to 95)	<0.0001
			37 (1 to 243)	17 (1 to 95)	<0.0001
Specialist	55	275	−17 (−95 to 241)^*^	13 (−59 to 143)	<0.0001
			32 (o to 241)	19 (1 to 143)	<0.0001
*p*			^*^<0.05	^*^0.0128	

* *Denotes significance between groups. Note: for comparison between the PGD groups for the 126 data sets retained in the Comparison phase the significant difference between Practitioner and Specialist groups was retained, however the p-value was different*.

#### Scenarios

The raw estimation values between the PGD and WGD phases were different for all of the scenarios. Scenario 1 (*p* < 0.0001), and Scenario 2 (*p* < 0.0001), showed an increased median error in the WGD phase compared with the PGD phase, while Scenarios 3 (*p* < 0.0001), 4 (*p* < 0.0001) and 5 (*p* < 0.0001) showed a reduced median error in the WGD phase compared with the PGD phase.

For the absolute estimation error, Scenarios 1 and 2 were not different. Scenarios 3 (*p* < 0.0001), 4 (*p* < 0.0001), and 5 (*p* < 0.0001) were different and had a reduced median absolute estimation error between the PGD and WGD phases. All results are displayed in Table 3.

**Table 3:** Scenario results displayed in mL as median and range for Pre-Guide (PGD) and With-Guide (WGD) as raw and absolute values.

**Scenario**	**Actual volume (mL)**	**PGD 126 (raw)**	**WGD (raw)**	***p***
		**PGD 126 (absolute)**	**WGD (absolute)**	
1	66	−16 (−56 to 114)	34 (−56 to 114)	<0.0001
		34 (1 to 114)	34 (1 to 114)	0.5395
2	105	−5 (−95 to 95)	35 (−80 to 95	<0.0001
		30 (5 to 95)	35 (5 to 95)	0.8699
3	57	−37 (−54 to 243)	3 (−50 to 93)	<0.0001
		37 (2 to 243)	19 (0 to 93)	<0.0001
4	57	−22 (−52 to 143)	8 (−52 to 143)	<0.0001
		27 (0 to 143)	13 (0 to 143)	<0.0001
5	109	−34 (−99 to 241)	1 (−79 to 141)	<0.0001
		49 (1 to 241)	11 (1 to 141)	<0.0001

## Discussion

### Pre-guide Accuracy

Our primary hypothesis that visual estimation would be inaccurate was confirmed. This finding is in line with both medical and veterinary research which found underestimation to be common (4, 8, 14, 16–18). However, contrary to medical literature which found smaller volumes to be overestimated, the relatively small volumes in this study were underestimated (19, 20). There has been speculation that lack of formal training contributes to estimation inaccuracy (20, 21).

In this study the error and range is smaller in this population compared with an earlier phase of the same study which surveyed a more diverse population at a single university institution (14). This is similar to the findings of a previous study which found that anesthetists were the most accurate in a population which included obstetricians and nurses (22). This potentially reflects the greater experience of this population with blood estimation.

This is an important finding as hemorrhage has broad-reaching consequences including impairment of circulation, compromised oxygen delivery and extraction, and, ultimately, reduced aerobic metabolism (23–25). Fluid therapy decisions to manage hypovolaemia should aim to restore circulation and perfusion while maintaining adequate oxygen delivery (26). Accurate categorization of severity and adequacy of response is critical to ensuring optimal outcomes (24). Similarly, overzealous fluid administration can be detrimental and waste finite resources (27, 28). Physiologic parameters such heart rate and systolic blood pressure have traditionally been used to evaluate circulatory competency and the impact of hemorrhage and hypovolaemia (29, 30). However, both in the medical and veterinary literature, these parameters have been shown to be both slow to indicate early hemorrhagic shock and prone to inconsistency (3, 29–35). Even dPP, though more sensitive than other physiological parameters, is sensitive only with 5% or greater blood loss (7). Similarly, laboratory parameters such as elevated lactate tend to lag behind the occurrence of hypoperfusion (2). In the medical literature, the relationship between objective measures such as hemoglobin concentration and mortality, and its use to inform transfusion have been described (34, 36). Studies in the veterinary literature have shown a weaker relationship for these measures, further suggesting that reliance on late-changing systemic parameters could result in poorer patient outcomes and emphasizing the importance of accurate estimation of blood loss (37).

Our results indicated no difference between the role groups. This is contrary to Ashburn et al. (38) who found that attending physician had a reduced error compared with residents. Our finding is, however, similar to Adkins et al. (5) who investigated groups of anesthesia providers and found no difference between different roles and levels of experience and expertise. If the role is considered a proxy for expertise, this suggests that, in the field of veterinary anesthesia, expertise has no effect on the ability to visually estimate blood volumes. This may be due to a lack of specific training on blood volume estimation regardless of role level. Alternatively, it may suggest that anesthesia staff are similarly competent at estimating blood volumes, reflecting a similar work-interest as reported in the medical literature (39).

The scenarios were constructed with increasing complexity, though not necessarily increasing volume, as they progressed. Each subsequent scenario had more items to be estimated, less easily estimable volumes, or a combination of both, than the former. Therefore, it would be logical to expect that the more complex scenarios would incur the greatest error. The results largely confirmed this hypothesis, with PGD differences between Scenarios 3, 4, and 5 and increasing error with each successive scenario, for both the raw error and the absolute error. The only instance where this trend was not followed was Scenario 3 which showed a greater raw error than Scenario 4 and was not different to Scenario 5. This is consistent with previous similar work at a single university institution which also saw a significant underestimation of the actual volume for the same scenario (14). The scenario consisted of a puddle and single 10 × 10 cm swab and may suggest that moderate puddles and small surgical items, may present a significant estimation challenge.

The scenarios did not show a relationship between accuracy and volume. This contrasts medical literature where one study showed smaller volumes had better accuracy than larger volumes, and another study showed smaller volumes were overestimated and larger volumes were underestimated (16, 19). The lack of correlation of accuracy and volume as well as the tendency toward underestimation seen in the current study may represent the effect of the veterinary perspective of the participants. Even though the volumes are necessarily smaller to reflect the veterinary situation, the underestimation finding is consistent with the majority of the medical literature and suggest that this may be associated with the environment and experience of the respondent rather than the actual volume *per se*.

### With-Guide Accuracy

Accuracy improved for estimations using the pictorial guide, shifting from underestimation to overestimation, with a narrower range. This is consistent with similar studies in the medical literature who found that estimation improved with training or education (20, 39). Larsson et al. (40) found that established guidelines for estimation reduced the inter-observer variation, and this is seen in the current study with the narrowed WGD range compared to PGD.

In comparison with the previous single institution study of the same design, these results provide greater evidence of the utility of the Guide. In the previous study the absolute error was reduced, but the raw error was increased from the PGD phase to the WGD phase. While the utility of the Guide was supported by a narrower range, the median error increase in the previous study meant that a universal application could not be concluded.

All of the role groups had a reduced error for both raw and absolute values for WGD compared with PGD phases (*p* < 0.001). This is more comprehensive evidence of the ability of the Guide to improve estimation accuracy. In the previous study, several role groups saw an increase in raw error, and/or a statistically non-significant decrease in absolute error. The reduction in the raw estimation as well as the magnitude of the error across all role groups supports the conclusion that a pictorial Guide is useful for a variety of roles and experience, irrespective of training level.

The Guide assisted in reducing the raw and absolute estimation error for the more complex Scenarios 3, 4, and 5 (*p* < 0.0001). In the WGD phase there were no differences between these scenarios, suggesting that the Guide reduced the variable effect of multiple elements of scenario complexity seen in the PGD phase. In particular, Scenarios 3 and 5 which had the greatest PGD error saw a large reduction in error which supports the utility of the Guide in challenging situations. With a greater variety of blood collection receptacles and spilled volumes, these scenarios are likely to be more reflective of real-life surgical situations and as such support the utility of the Guide in clinical practice. As seen in the previous study, the less complex scenarios resulted in an increase in the raw error and no change in the absolute error. It is possible that this was a bias introduced into the results by the study design which used very similar images for the simple scenarios (1 and 2) to those used in the Guide. This may have resulted in a respondent short-cut where the inputted WGD estimation was the same value exactly as the similar image from the Guide, causing a greater over-estimation error.

There are several limitations in this study. The survey and presented scenarios were online images only, meaning that direct assessment of the blood volumes as would be possible in the clinical situation could not occur, potentially reducing the accuracy of the responses. Artificial blood was used to create the images and despite a gross similarity to actual blood, this may have introduced assessment error. Although steps were taken to assess and confirm data quality, or eliminate poor data, as this was a remote online survey only, it is impossible to be entirely confident of each data point. The scenarios were limited to small animals only and the results cannot be extrapolated to species other than dogs and cats. However, as seen in other studies, estimation accuracy often declines with increasing blood volumes (8). Similarly, with respect to the companion animal focus, the blood volumes were low and the variation between the scenarios was comparatively small, making differences between the groups or scenarios less obvious. The use of similar images in both the scenarios and the Guide may mean that the utility of the Guide is limited to assessment of similar materials in a clinical situation and may not translate to dissimilar materials. A degree of learning through repetition of the scenarios across the phases may have confounded the results, and this effect was not tested with a sample of respondents participating without the pictorial guide. The scenarios were not randomized as it was considered by the investigators that this would not affect the outcome, but may have contributed to learning. Although the overall number of respondents was in excess of the requirement indicated by the sample size calculation, the sample size of the role groups may have been insufficient to achieve statistical significance for evaluation between groups. Finally, the sample population was limited to veterinarians and associates with a focus on anesthesia, and as such wider conclusions for broader-discipline populations may not be possible.

In conclusion, visual estimation of blood loss is inaccurate, decreases with increasing scenario complexity, and there are no differences between different groups according to role, gender, experience, or country of origin. A pictorial guide improves the accuracy for scenarios involving small animals for anesthesia-focused veterinarians and associates. This guide is most useful in complex scenarios involving more than one type of receptacle for blood collection. Further work to assess the utility of a pictorial guide in a clinical situation is required.

## Data Availability Statement

The raw data supporting the conclusions of this article will be made available by the authors, without undue reservation.

## Ethics Statement

The studies involving human participants were reviewed and approved by The University of Sydney Human Research Ethics Committee (HREC). The patients/participants provided their written informed consent to participate in this study.

## Author Contributions

SC: idea development, study design, data and statistical analysis, and manuscript preparation and writing. FM-T: idea development, study design, data and statistical analysis, manuscript preparation and revision, and overall supervision of work.

## Conflict of Interest

The authors declare that the research was conducted in the absence of any commercial or financial relationships that could be construed as a potential conflict of interest.
