# Supplementary material for: Blood Loss Estimation in Small Animals and Assessment of a Pictorial Tool to Improve Accuracy in a Global Population of Veterinary Anesthesia Staff
Source: Front Vet Sci. 2020 May 7;7:212. doi: 10.3389/fvets.2020.00212 (PMC7221018; doi:10.3389/fvets.2020.00212)
Supplement: Supplementary file 2 [file Data_Sheet_1.PDF]

The aim of this survey is to evaluate the accuracy of estimation of blood loss during surgical procedures with and without the use of a guiding tool. Your participation in this survey is completely voluntary and you may withdraw at any time.

Completing this only survey will take no more than 10 minutes. All data is stored in a password protected electronic format. The survey and data collected will not contain any personally identifying information such as your name, email address or IP address. All obtained data will be used only for research purposes. The data will be published.

A Participant Information Sheet is available here.

Please click 'OK' to proceed to the agree/disagree page.
By selecting 'I agree to participate' button, you are indicating that your are at least 18 years old, have read and understood the information presented above and voluntarily agree to participate in the survey. If you do not wish to participate then please select 'I do not wish to participate'.

OK

* 1. Do you agree to participate in the survey?

- ◯ Yes, I have read the above information and agree to participate in the survey.
- ◯ No, I do not wish to participate in the survey.

* 2. Which country are you completing this survey from?

* 3. What is your professional role/level of expertise?

- ◯ Nurse/Technician
- ◯ Student
- ◯ General Practice Veterinarian
- ◯ Advanced General Practice Veterinarian (Membership/Certificate - any discipline)
- ◯ Intern
- ◯ Resident/ Residency Trained
- ◯ Specialist (e.g. European or American Diplomat, ANZCVS Fellow)

Other (please specify)

4. How many years of experience do you have?

- ◯ <2 years
- ◯ 2-5
- ◯ 5-10
- ◯ 10-15
- ◯ >15
- ◯ I am still a student

5. Are you:

- ◯ Female
- ◯ Male
- ◯ Prefer not to say

* 6. Scenario 1 - A 5 kg cat undergoing a cystotomy.

What is your estimate of the volume of blood (in mL) contained in the image of the material below? (please give your answer as a single number only)

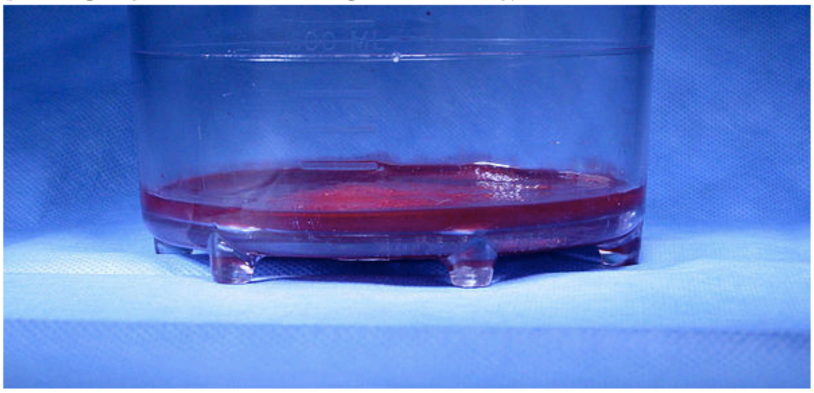

* 7. Scenario 2 - A 10kg dog undergoing a Tibial Plateau Leveling Osteotomy (TPLO).

What is your estimate of the volume of blood (in mL) contained in the image of the material below? (please give your answer as a single number only)

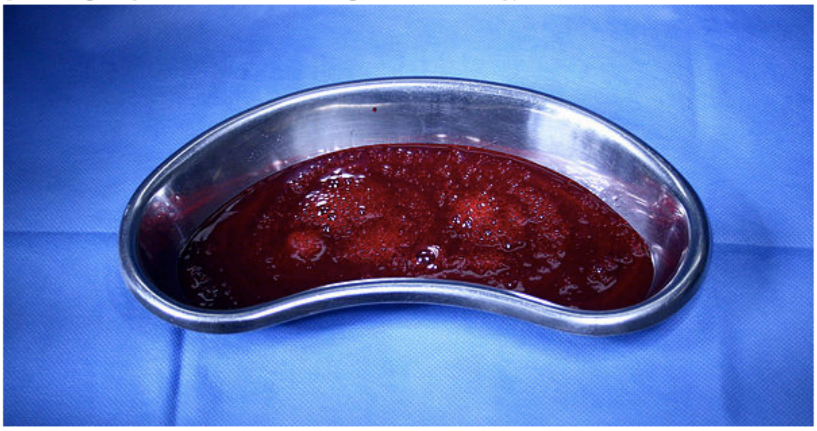

(500 mL kidney dish)

* 8. Scenario 3 - A 4.5 kg cat undergoing a perineal urethrostomy.

What is your estimate of the **combined total** volume of blood (in mL) contained in the images of the material below?
(please give your answer as a single number only)

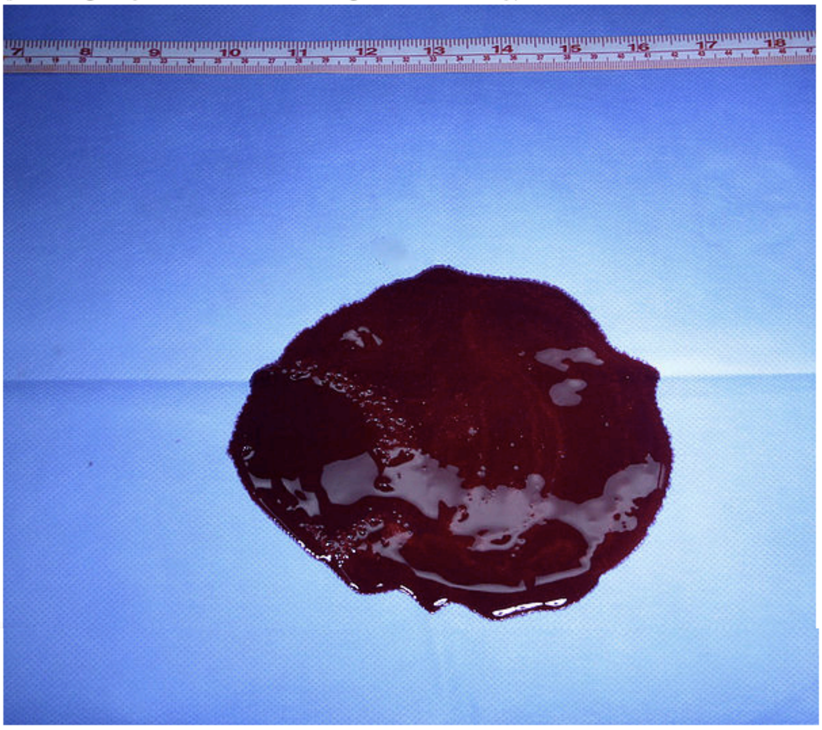

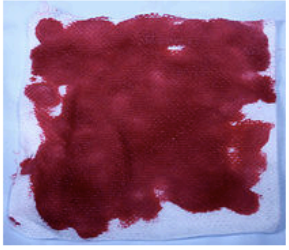

(10 cm x 10 cm gauze swab)

* 9. Scenario 4 - A 15 kg dog undergoing an ovariohysterectomy (spey).

What is your estimate of the **combined total** volume of blood (in mL) contained in the images of the material below?
(please give your answer as a single number only)

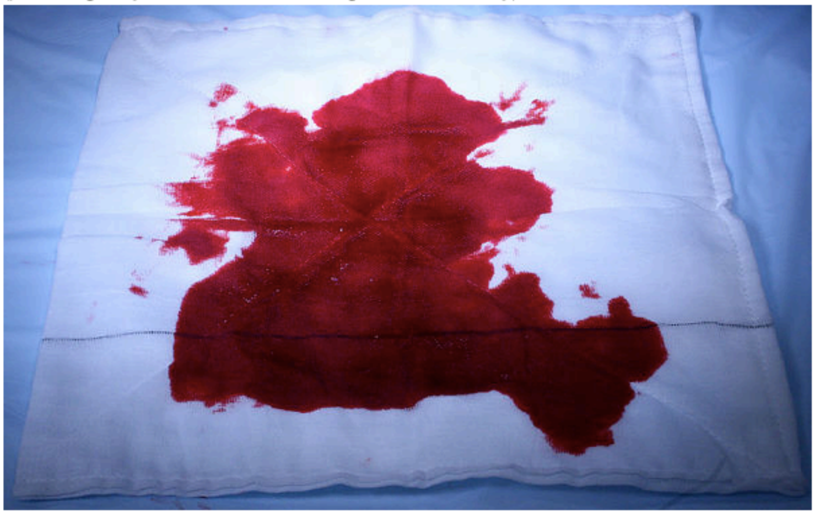

(30 cm x 30 cm laparotomy sponge)

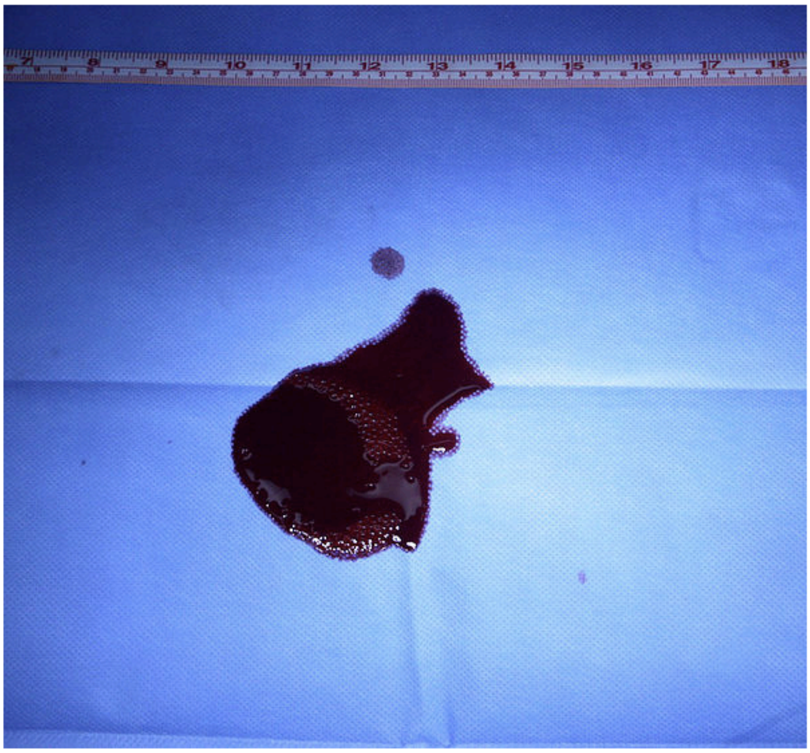

* 10. Scenario 5 - A 12 kg dog undergoing a splenectomy.

What is your estimate of the **combined total** volume of blood (in mL) contained in the images of the material below?
(please give your answer as a single number only)

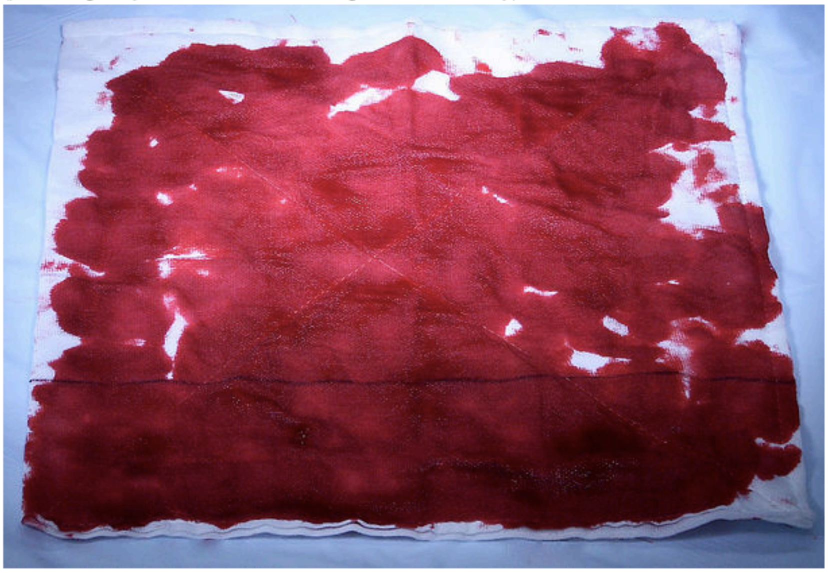

(30 cm x 30 cm laparotomy sponge)

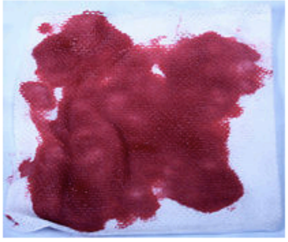

(10 cm x 10 cm gauze swab)

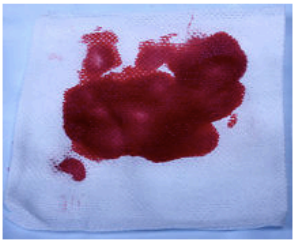

(10 cm x 10 cm gauze swab)

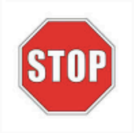

Before proceeding to the next question:

CLICK ON THIS LINK and examine The Guide.

(Note: Open the link as a new tab, keep the page open and come back to this survey)

The Guide has been created as a reference tool to help in estimation of blood loss. **Please keep the page open. You may consult this guide as often as you like and in conjunction with your estimations for the following questions.**

OK

* 11. Did you follow the link above?

- Yes, the Guide is ready to help me with the next section.
- No. I may need to go back.

* 12. Scenario 1 - A 5 kg cat undergoing a cystotomy.

What is your estimate of the volume of blood (in mL) contained in the image of the material below? (please give your answer as a number only). **Remember you can use the Guide to help you.**

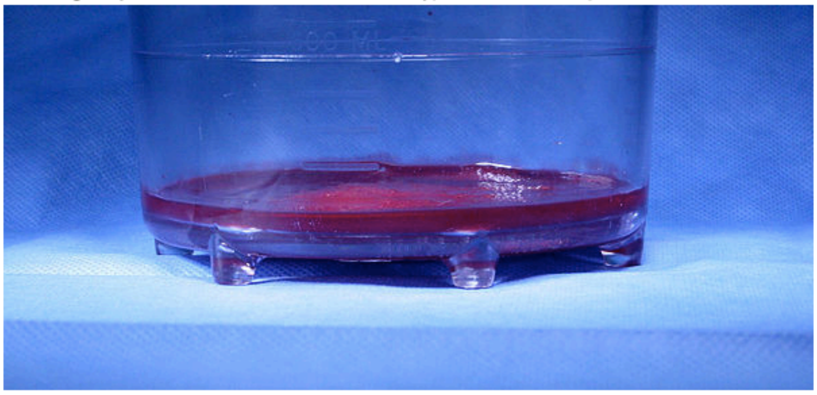

* 13. Scenario 2 - A 10kg dog undergoing a Tibial Plateau Leveling Osteotomy (TPLO).

What is your estimate of the volume of blood (in mL) contained in the image of the material below? (please give your answer as a number only) **Remember you can use the Guide to help you.**

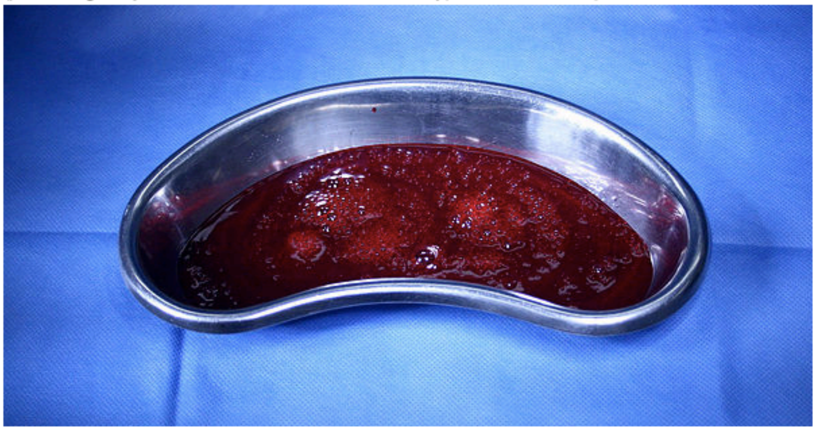

(500 mL kidney dish)

* 14. Scenario 3 - A 4.5 kg cat undergoing a perineal urethrostomy.

What is your estimate of the **combined total** volume of blood (in mL) contained in the images of the material below?
(please give your answer as a single number only). **Remember you can use the Guide to help you.**

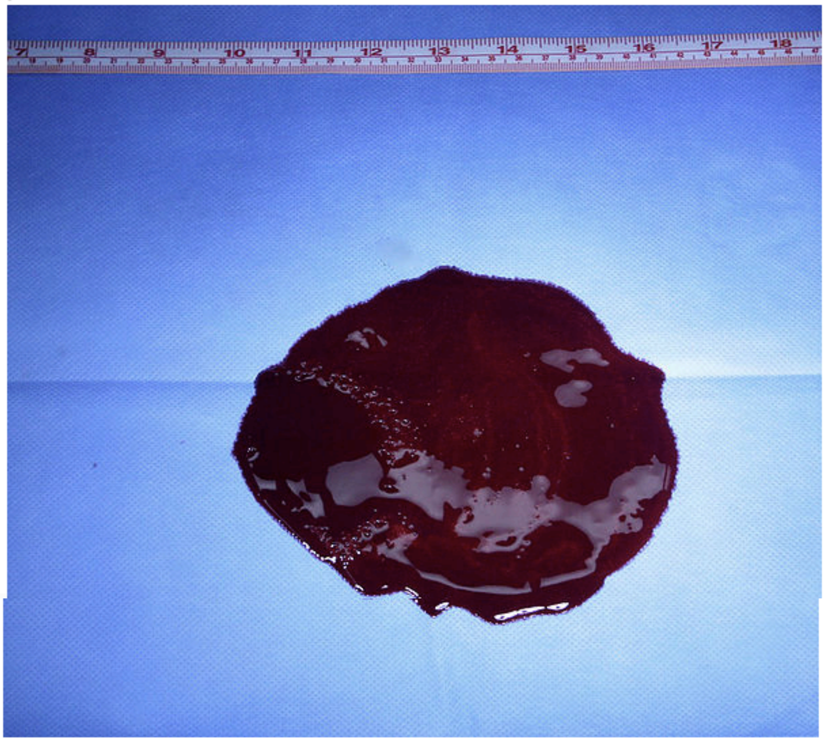

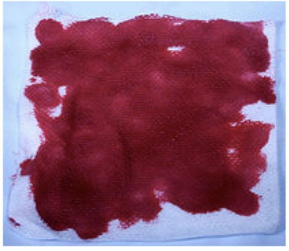

(10 cm x 10 cm gauze swab)

* 15. Scenario 4 - A 15 kg dog undergoing an ovariohysterectomy (spey).

What is your estimate of the **combined total** volume of blood (in mL) contained in the images of the material below?
(please give your answer as a single number only). **Remember you can use the Guide to help you.**

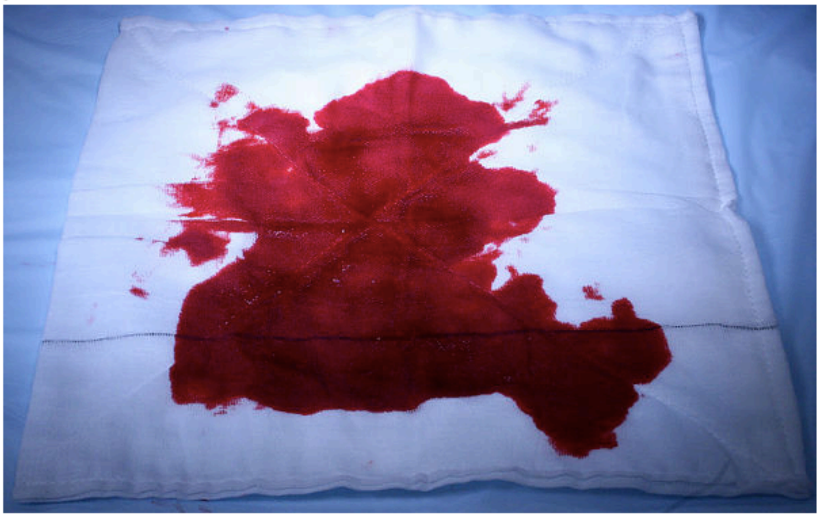

(30 cm x 30 cm laparotomy sponge)

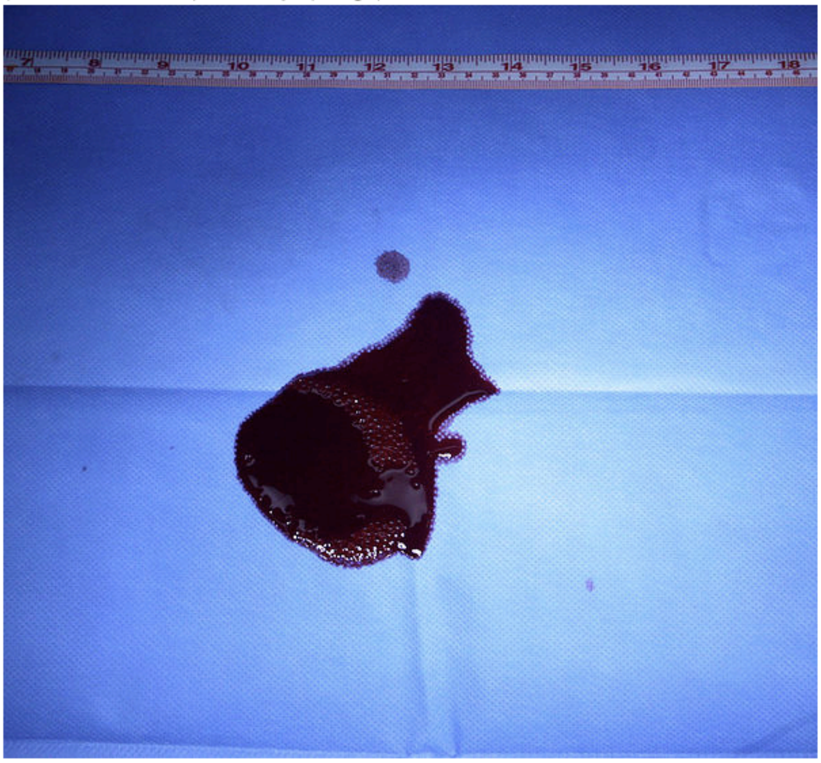

* 16. Scenario 5 - A 12 kg dog undergoing a splenectomy.

What is your estimate of the **combined total** volume of blood (in mL) contained in the image of the material below?
(please give your answer as a single number only). **Remember you can use the Guide to help you.**

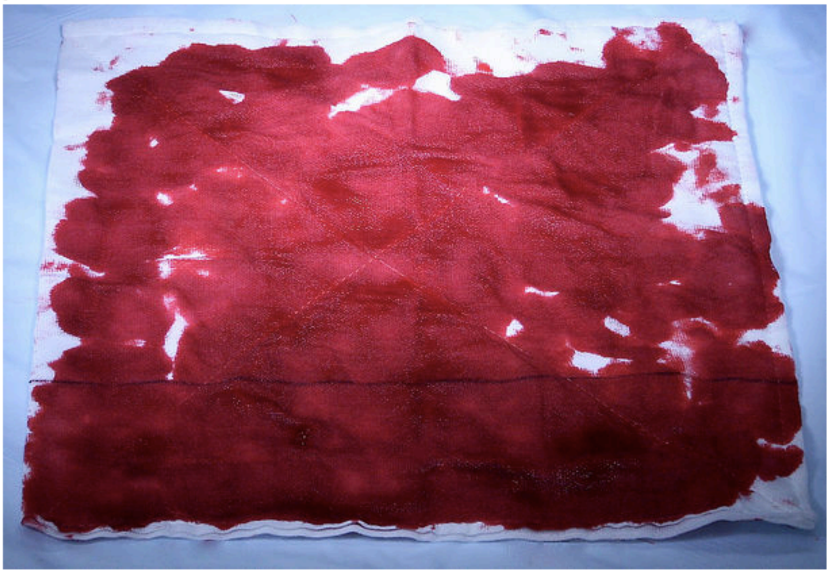

(30 cm x 30 cm laparotomy sponge)

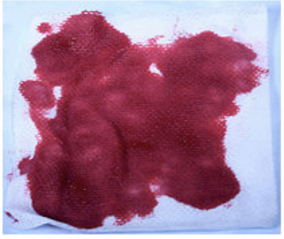

(10 cm x 10 cm gauze swab)

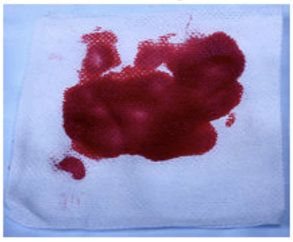

(10 cm x 10 cm gauze swab)
