# Supplementary material for: Blood Loss Estimation in Small Animals and Assessment of a Pictorial Tool to Improve Accuracy in a Global Population of Veterinary Anesthesia Staff
Source: Front Vet Sci. 2020 May 7;7:212. doi: 10.3389/fvets.2020.00212 (PMC7221018; doi:10.3389/fvets.2020.00212)
Supplement: Supplementary file 3 [file Data_Sheet_2.PDF]

This site was designed with the WiX.com website builder. Create your website today. Start Now

Blood Loss Estimation

The Guide

Please return to the survey when you have finished examining this Guide

2 L Suction Pot

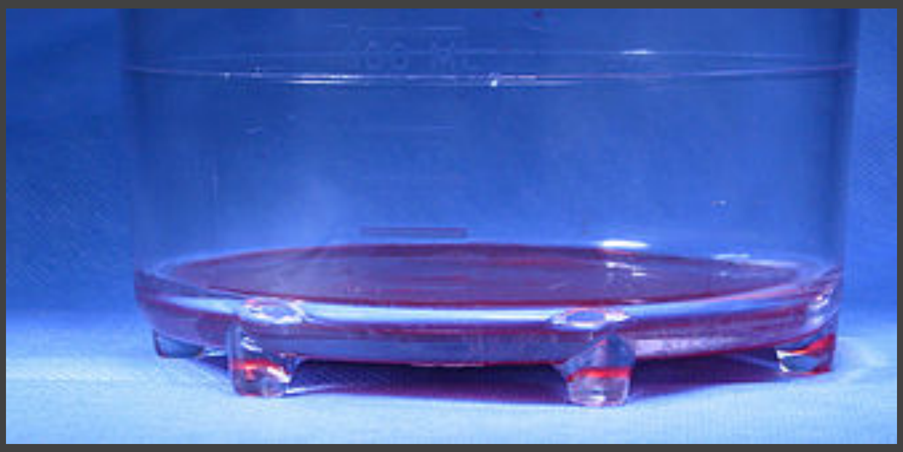

40 mL

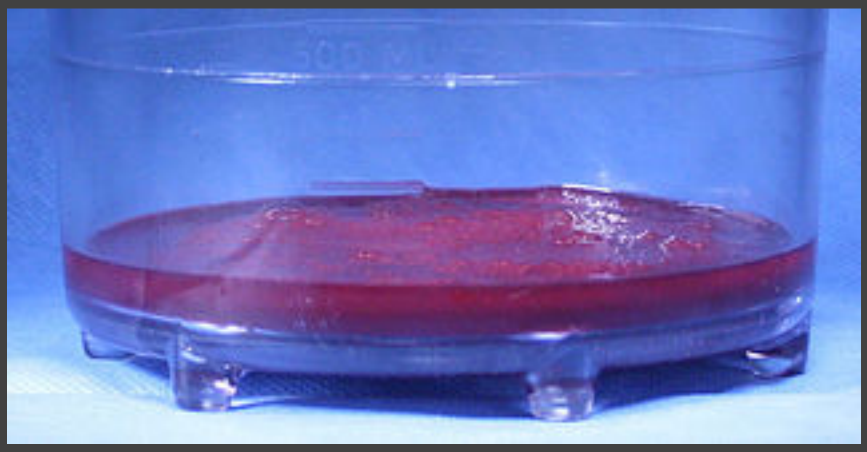

120 mL

500 mL Kidney Dish

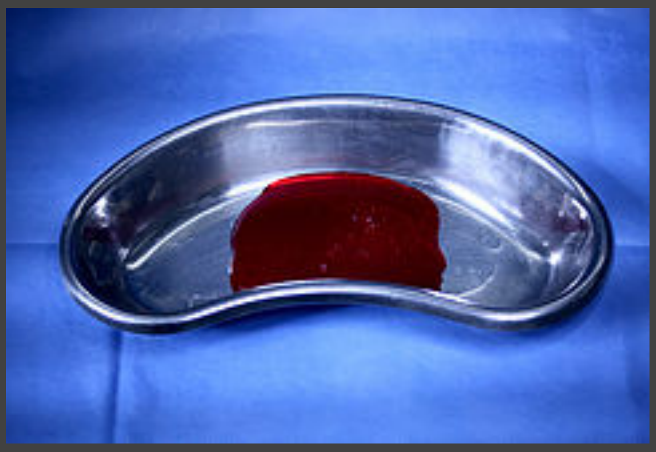

20 mL

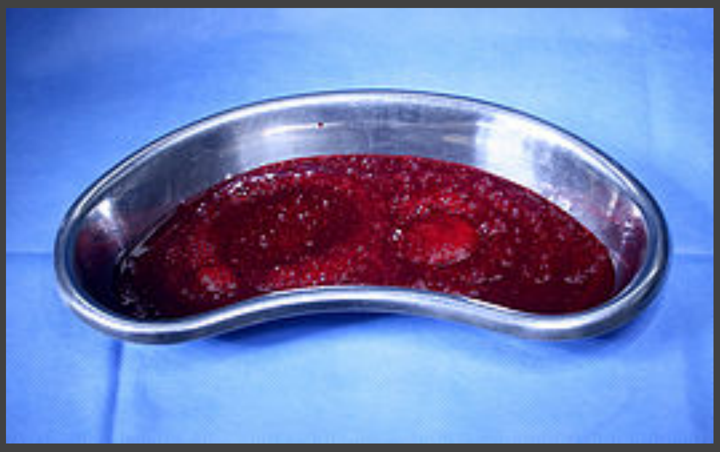

140 mL

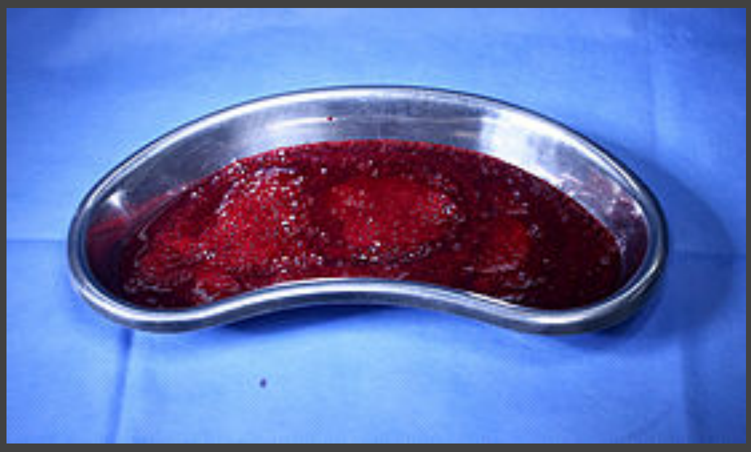

200 mL

Lap Sponge 30 x 30 cm

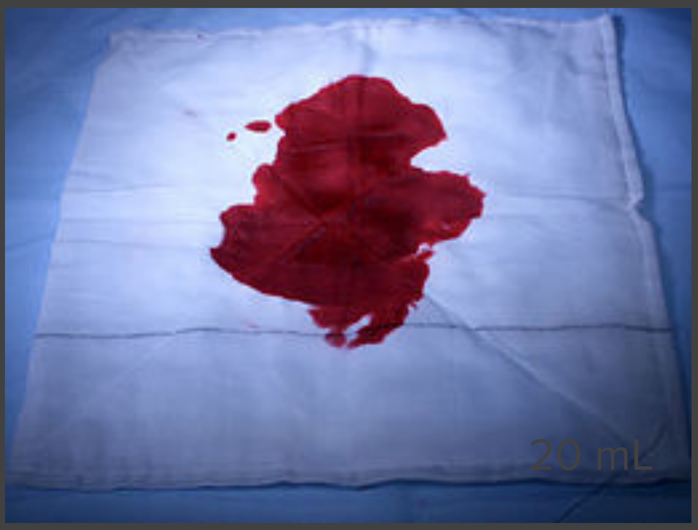

20 mL

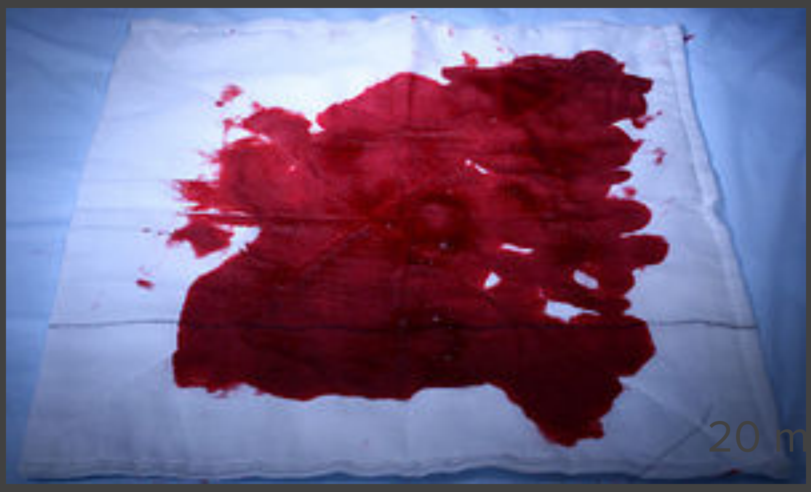

60 mL

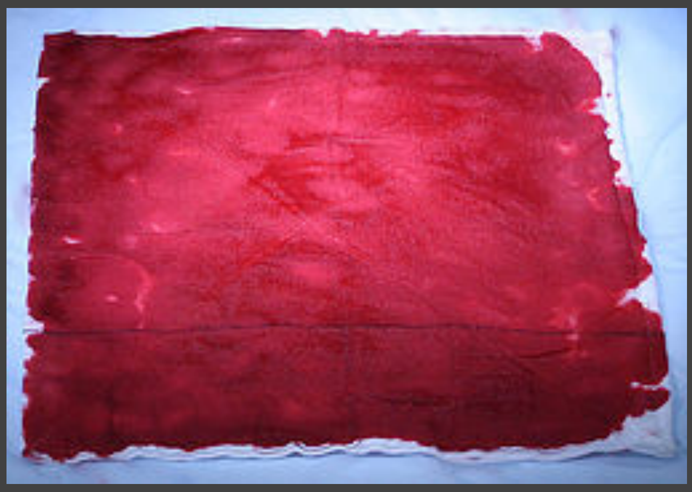

120 mL

Swab 10 x 10 cm

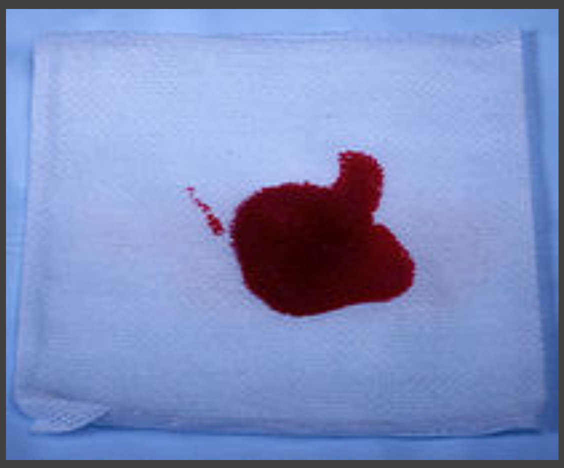

1 mL

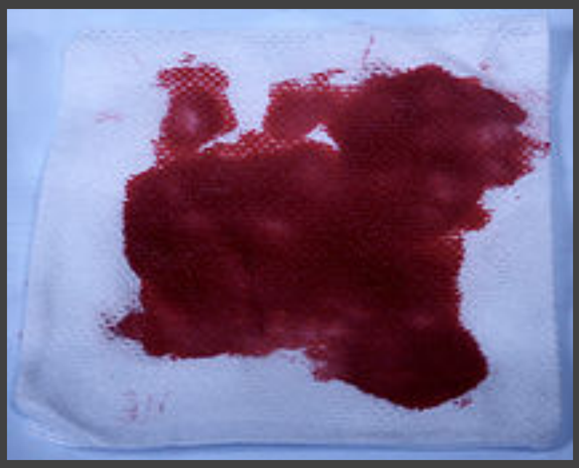

4 mL

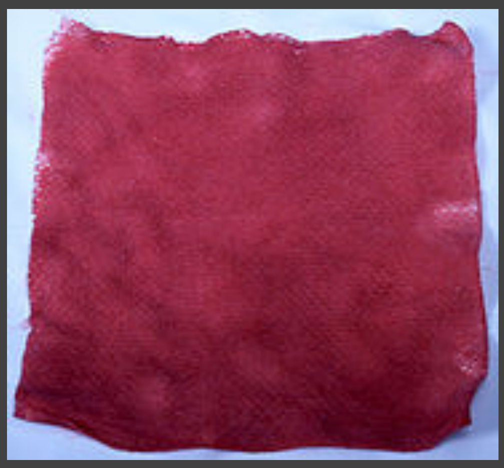

8 mL

Puddle

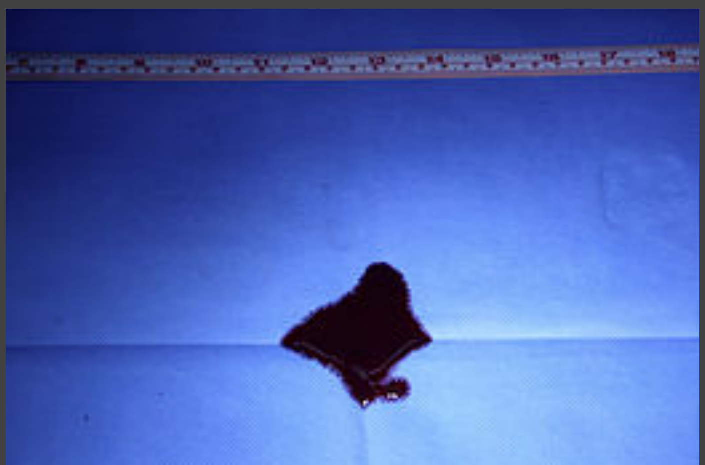

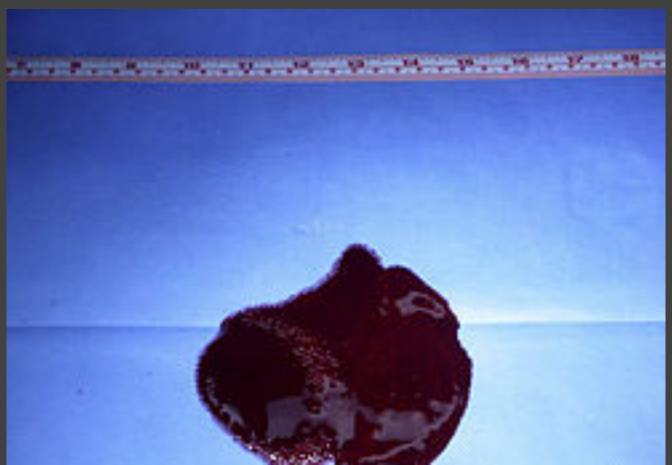

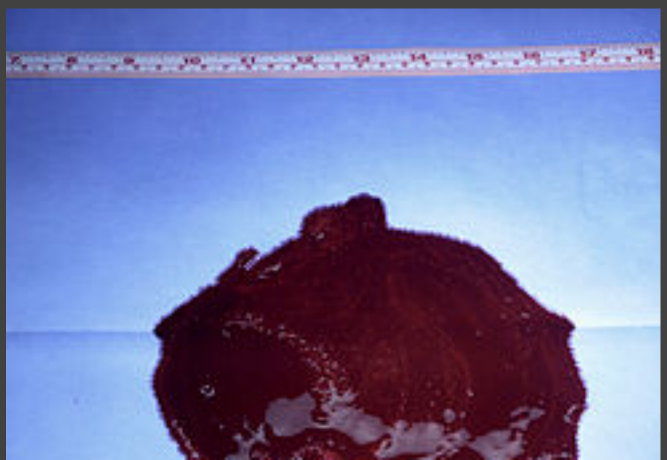

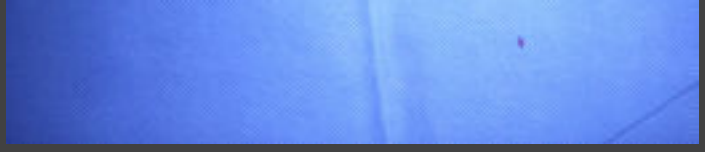

5 mL

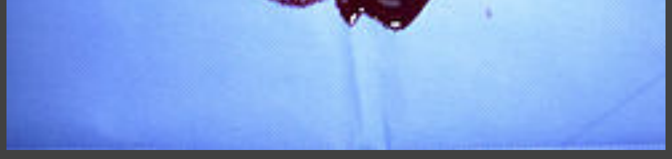

30 mL

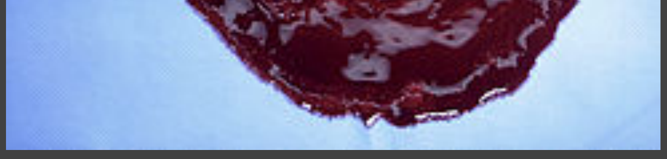

70 mL

Please return to the survey when you have finished examining this Guide
